# NURSE WAGES IN NURSING HOMES: ANTECEDENTS AND CONSEQUENCES

**DOI:** 10.1093/geroni/igae098.0533

**Published:** 2024-12-31

**Authors:** Rohit Pradhan, Jane Banaszak-Holl

**Affiliations:** Chair; Discussant

## Abstract

A substantial body of research has established that nurse staffing intensity and skill mix have a consequential impact on nursing home (NH) care quality. Unfortunately, NH staffing has been marked by multitudes of challenges including high levels of turnover and absenteeism. The literature suggests that an important reason why NHs have struggled with nurse staffing is the relatively lower wages vis-à-vis other important areas of the U.S. healthcare system, such as hospitals. These challenges underscore the importance of examining nurse wages within the NH setting, as wages not only affect staff retention and satisfaction but overall performance. Despite the apparent need for competitive wages to attract and retain skilled nurses, NHs operate in a resource-constrained environment, making wage increases a complex issue for administrators. The overarching goal of this symposium is to explore the antecedents and consequences of nurse wages in NHs. As part of the symposium, we include studies examining nurse wages and quality, the organizational and market-level factors associated with nurse wages, and the impact of nurse wages on financial performance. The findings and discussions may offer valuable insights for both managerial decision-making and policy formulation, aiming to address the systemic challenges of nurse staffing in NHs and improve NH performance, particularly quality of care.

